# Biogeographic history and high-elevation adaptations inferred from the mitochondrial genome of Glyptosternoid fishes (Sisoridae, Siluriformes) from the southeastern Tibetan Plateau

**DOI:** 10.1186/s12862-015-0516-9

**Published:** 2015-10-28

**Authors:** Xiuhui Ma, Jingliang Kang, Weitao Chen, Chuanjiang Zhou, Shunping He

**Affiliations:** School of Life Science, Southwest University, Beibei, Chongqing, 400715 China; The Key Laboratory of Aquatic Biodiversity and Conservation of Chinese Academy of Sciences, Institute of Hydrobiology, Chinese Academy of Sciences, Wuhan, Hubei 430072 China; University of Chinese Academy of Sciences, Beijing, 10001 China

**Keywords:** Glyptosternoids, High-elevation environment, Mitochondrial genome, PCR-based next-generation sequencing, Southeastern Tibetan drainage patterns

## Abstract

**Background:**

The distribution of the Chinese Glyptosternoid catfish is limited to the rivers of the Tibetan Plateau and peripheral regions, especially the drainage areas of southeastern Tibet. Therefore, Glyptosternoid fishes are ideal for reconstructing the geological history of the southeastern Tibet drainage patterns and mitochondrial genetic adaptions to high elevations.

**Results:**

Our phylogenetic results support the monophyly of the Sisoridae and the Glyptosternoid fishes. The reconstructed ancestral geographical distribution suggests that the ancestral Glyptosternoids was widely distributed throughout the Brahmaputra drainage in the eastern Himalayas and Tibetan area during the Late Miocene (c. 5.5 Ma). We found that the Glyptosternoid fishes lineage had a higher ratio of nonsynonymous to synonymous substitutions than those found in non-Glyptosternoids. In addition, ω_pss_ was estimated to be 10.73, which is significantly higher than 1 (*p*-value 0.0002), in COX1, which indicates positive selection in the common ancestral branch of Glyptosternoid fishes in China. We also found other signatures of positive selection in the branch of specialized species. These results imply mitochondrial genetic adaptation to high elevations in the Glyptosternoids.

**Conclusions:**

We reconstructed a possible scenario for the southeastern Tibetan drainage patterns based on the adaptive geographical distribution of the Chinese Glyptosternoids in this drainage. The Glyptosternoids may have experienced accelerated evolutionary rates in mitochondrial genes that were driven by positive selection to better adapt to the high-elevation environment of the Tibetan Plateau.

**Electronic supplementary material:**

The online version of this article (doi:10.1186/s12862-015-0516-9) contains supplementary material, which is available to authorized users.

## Background

The Tibetan Plateau (the “Roof of the World”) is the highest plateau on earth, with an average elevation of more than 4000 m. The plateau, which covers more than 2,500,000 km of plateaus and mountains in central Asia and is surrounded by towering mountain ranges, has been designated as a global hotspot of biodiversity [1]. The environment of the Tibetan Plateau is characterized by hypoxia and low temperatures [2]. Despite its inhospitable environment, various adaptive responses that may be responsible for highland adaptation have been identified in several species, including Tibetans [3–7], yak [8], Tibetan antelope [9], Tibetan wild boar [10], ground tit [11], Tibetan mastiff [12, 13], and a schizothoracine fish [14]. Among these adaptive processes, genes exhibiting signs of positive selection and expansion were significantly enriched in hypoxia and energy metabolism pathways. Mitochondrion plays an essential role in ATP synthesis and heat generation, and intense selection pressures may preferentially affect mitochondria in high-elevation environments. Previous studies have detected signals of positive selection in the mitochondrial genomes of organisms living at high elevations, including goats [15], Tibetan antelope [16], Tibetan asses [17], Tibetan horses [18], pikas [19], Chinese snub-nosed monkeys [20], and bar-headed geese [21]. However, most of these studies have focused on mammals or birds. Among fish, only the high-elevation adaptions of the schizothoracine fishes (Cyprinidae) have been examined [22].

Glyptosternoids refer to catfishes in the family Sisoridae subfamily Glyptosterninae tribe Glyptosternina. Currently, there are around 10 genera and 71 species of glyptosternoids, which 9 genera and 31 species distributed in China (http://www.calacademy.org/scientists/projects/catalog-of-fishes). Chinese Glyptosternoids are found in the rivers around the Tibetan Plateau and eastern Himalayas, e.g., the Yaluzangbujiang (Brahmaputra River), Irrawaddy, Nujiang (Upper Salween), Lancangjiang (Upper Mekong River), Jinshajiang (Upper Yangtze), Yuanjiang (Red River), Nanpanjiang (Upper Pearl River) and the Brahmaputra basin [23]. The Glyptosternoids (Siluriformes) represent one of the three broad fish lineages (including the schizothoracines and *Triplophysa*) commonly found on the Tibetan Plateau. Habitat is thought to play a crucial role in diversification, and changes in habitat likely affect the distribution and diversification of biota in a particular region [24]. In turn, the historical biogeography of a lineage reflects aspects of the history of the region in which the species, or lineage, is distributed. The collision between India and Asia caused the uplift of the Tibetan Plateau in the Late Eocene [25, 26], which affected the fauna (e.g., fish [23, 27, 28], frogs [29] and pikas [30]), the climate [31], and the rivers in this region [32]. Chinese Glyptosternoids provide an excellent resource with which to infer the geological and environmental history of the region. Several studies have investigated the phylogeny, biogeography and evolution of the Glyptosternoids [23, 28, 33–35]. Due to the unique distribution and morphology of the fishes of this lineage, the relationships between the speciation, evolution and biogeography of these species and the Tibetan Plateau has become an area of intense research [28, 33, 34, 36–38].

Three different explanations have been suggested for the extant distribution patterns of the Chinese Glyptosternoids. (1) Hora and Silas suggested that the Glyptosternoids originated in the eastern Himalayan area of Yunnan province, southwestern China, but the exact origin and route of expansion were not clear [36]. (2) Based on the fossil records of *Bagarius yarrelli*, Chu inferred that the Glyptosternoids originated in southeastern Tibet during the late Pliocene [39]. According to Chu, the *Glyptosternum*-like species then expanded eastward to western Sichuan and northern Yunnan after the formation of the Jinshajiang River. Diversification at the genus level was proposed to have occurred during the Pleistocene, with species then expanding into the rivers of Yunnan and Sichuan during the most recent uplift of the Himalayan area. (3) Other authors have suggested that the ancestor of the Glyptosternoids was widely distributed throughout the Tibetan Plateau in the early Pleistocene [33, 40] and that the ancestor of the *Glyptosternum*-like species maintained this distribution during the initial uplift of the Tibetan Plateau. The ancestor of the *Euchiloglanis*-like fish subsequently originated in the eastern Himalayan area during the second uplift of the Tibetan Plateau. *Euchiloglani*s-like species were then isolated to the Jinsha, Lancang, Nujiang, Yuanjiang, Pearl and Irrawaddy Rivers by the third uplift of the Tibetan Plateau. The specialized Glyptosternoids achieved their present distribution pattern due to the isolation of the rivers.

In this study, we aimed to reconstruct the ancestral distribution of the Glyptosternoids to test hypotheses concerning speciation with respect to southeastern Tibetan drainage patterns following the uplift of the Tibetan Plateau. Molecular clock approaches were used to infer divergence dates for this molecular phylogeny; to test whether the speciation, diversification and evolution of the Chinese Glyptosternoids are associated with the uplift of the Tibetan Plateau; and to examine high-elevation adaptive mitochondrial evolution in this lineage.

## Methods

### Muscle samples and DNA extraction

**Fig. 1:**
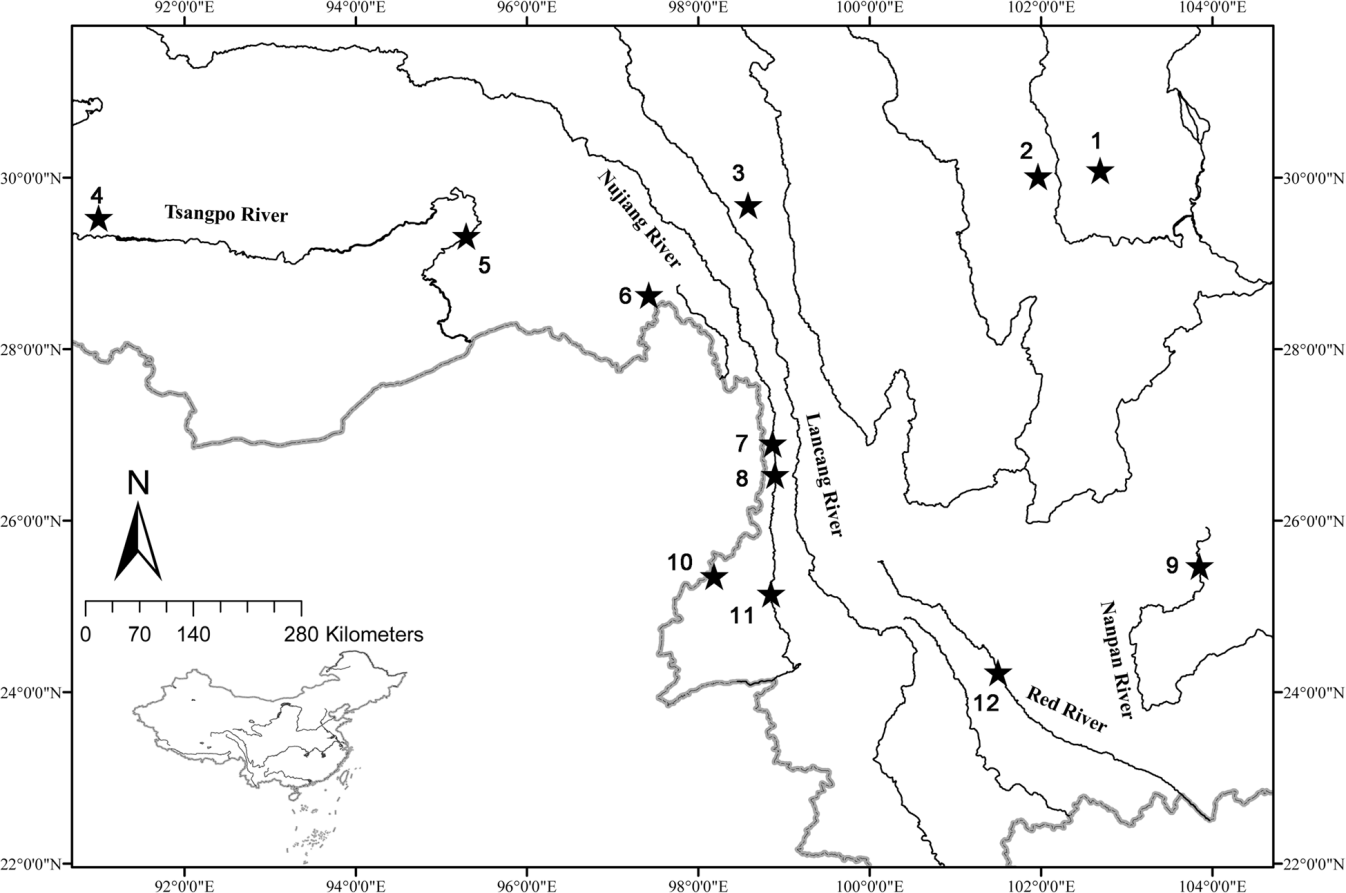
Geographic location of the studied Glyptosternoid fishes using ArcMap 9.1 software (ESRI Inc., Redlands, CA, USA). Details on the species are provided in Additional file 1: Table S1

The experiments were performed in accordance with the Ethics Committee of the Institute of Hydrobiology, Chinese Academy of Sciences. The Ethics Committee has also given ethics approval for our study. The policies were enacted according to Chinese Association for Laboratory Animal Sciences, and coordinated with the Institutional Animal Care and Use Committee (IACUC) protocols [41, 42]. The field work sample collection has also been permission according the Key Fund and NSFC-Yunnan mutual funds of the National Natural Science Foundation of China (Grant Nos. 31130049 and U1036603). Samples, including seventeen glyptosternoid species (18 individuals, more than half of Chinese glyptosternoids species), four other sisorids and three non-sisorids, following the system of Chu et al. [43] and references [44, 45], were collected from a variety of locations in China (Fig. 1 and Additional file 1: Table S1). As outgroups, *Liobagrus nigricauda* (Siluriformes: Amblycipitidae), *Cranoglanis bouderius* (Siluriformes: Cranoglanididae) and *Ictalurus punctatus* (Ictaluridae) included, putative close relatives to Sisoridae according to a recent study [46]. Voucher specimens were deposited at the Institute of Hydrobiology at the Chinese Academy of Sciences. Total genomic DNA was extracted from the muscle of a specimen using the OMEGA Genomic DNA Extraction Kit.

### Long PCR amplification

The complete mitochondrial genomes were amplified from the genomic DNA of the Sisoridae fishes using four overlapping amplification primers by long PCR methods: L9752 AGTACRAGTGACTTCCAATCACC, H2627 GTCCTGATCCAACATCGAGG, L295 GTAAAATTCGTGCCAGCCACC, and H10174 TCTGAGCCGAAATCAGAGGTC. The PCR reactions were prepared in total volumes of 50 μL as follows: 5x LongAmp buffer, 10 μL of each 2.5 mM dNTP, 6 μL of each 0.4 μM primer, 2.0 U LongAmp polymerase, and 20–50 ng of genomic DNA. The PCR conditions included an initial denaturation step at 94 °C for 30 s followed by 30–35 cycles at 94 °C for 30 s, 61–68 °C for 1 min, and 65 °C for 10 min, with a final extension of 10 min at 65 °C. Annealing temperatures were varied within these ranges in order to optimize the efficiency of different primers and samples.

### Library preparation

Library preparation was conducted using the “With-Bead” Method [47] with a slight modification: The Long PCR product was subsequently sheared to approximately 500 bp using a Covaris S2 Focused-ultrasonicator (Covaris, Inc., Woburn, Massachusetts, USA) before library preparation. The fragment sizes between 250 bp and 500 bp were selected by the gel extract method. After shearing, overhanging 5’- and 3’-ends were repaired by T4 DNA polymerase, 5’-phosphates were attached using T4 polynucleotide kinase, and P5 and P7 adapters were ligated to the ends of the repaired molecules using T4 DNA ligase. The resulting single-strand nicks are completed using Bst polymerase to allow amplification of the insert. The library was amplified using ‘off-bead amplification’ and tagged by different indexing primers.

### Quantifying and pooling library using q-PCR

We used the LightCycler 480 SYBR Green I Master (Roche, Basel, Switzerland) and qPCR Primers 1.1 and 2.1 according to the Illumina protocol (Illumina, USA). We pooled 40 indexed samples in equimolar ratios after they were quantified.

### Sequencing and assembly

We used 600 μL of 16 pM samples for paired-end 300 bp sequencing on a MiSeq sequencer (Illumina, Inc., San Diego, CA, USA). Sequence reads were sorted into each sample by the indices. As a first step, quality control checks were run on raw sequence data via fastqc (http://www.bioinformatics.bbsrc.ac.uk/projects/fastqc). The adapter sequences and sites with lower qualities were trimmed using Cutadapt [48] and the fastq_quality_filter tool (http://hannonlab.cshl.edu/fastx_toolkit/). Contigs were assembled *de novo* for each species using Trinity [49]. We then mapped all contigs to the mitochondrial genomes of their relative species one by one using LASTZ (available at http://www.bx.psu.edu/miller_lab/). Additional file 1: Table S2 provides the detailed characteristics of these mitochondrial genomes. The resulting consensus sequences were compared with sequences of ND2, D-Loop, and several other mitochondrial fragments generated from the same sample using independent Sanger sequencing.

### Phylogenetic analyses based on mitochondrial genomes

The original mitochondrial genome sequences of 10 species of Glyptosternoids were determined in this study, and the published mitochondrial genome sequences of 15 teleost species from GenBank were used to conduct phylogenetic analyses. *Cranoglanis bouderius*, *Ictalurus punctatus* and *Liobagrus nigricauda* were selected as outgroups. The accession numbers of all the sequences used in this study are summarized in Additional file 1: Table S1. Twelve protein-coding genes encoded in the heavy strand of DNA and two rRNA genes were used for the analyses. We excluded the ND6 gene because this gene is encoded on the light strand, and its nucleotide compositions are very different from other genes. Each gene sequence was automatically aligned using the MAFFT program [50] and carefully checked by eye. All ambiguous portions were excluded. After removing the start and stop codons, 12 protein-coding genes and 2 rRNA genes were concatenated.

We inferred the phylogenetic relationships via the maximum-likelihood (ML) method [51] and MrBayes software [52]. For the ML analyses, we used the RAxML program version 7.2.6 [53] with the general time reversible model with gamma distribution and a proportion of invariable sites (GTR + G + I) as estimated by Python programs (i.e., run mraic.py) [54]. Taking into account the different tempo and mode of the nucleotide substitutions, the parameters of the nucleotide substitution model and the branch lengths of the first, second, and third codon positions and ribosomal RNAs were separately estimated. To evaluate the confidence of the internal nodes, the rapid bootstrap method [53] was applied with 1000 replications. Using the MrBayes software, four independent chains were run for 10,000,000 generations with a burn-in length of 2500 generations and a sampling frequency of 1000 generations. Three of the four chains were heated, and the analysis was run twice.

### Reconstruction of ancestral geographical distribution

**Fig. 2:**
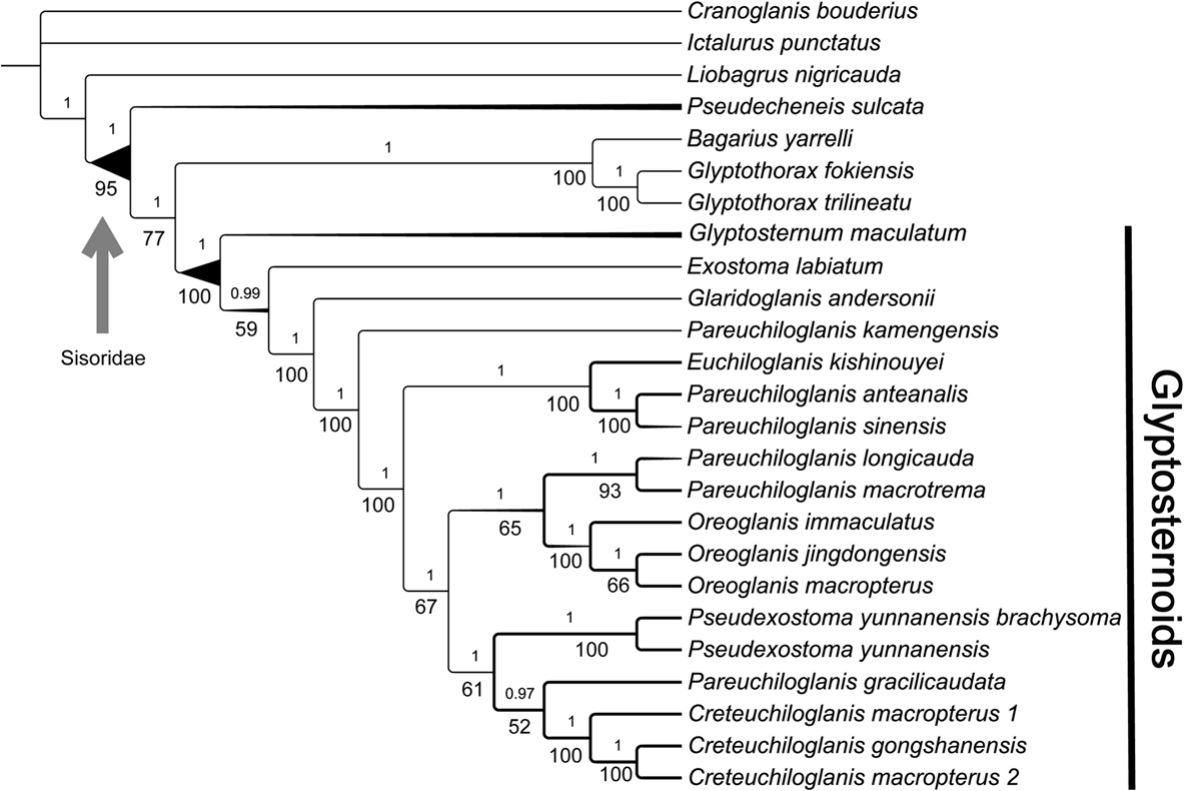
Phylogenetic tree estimated using the MrBayes algorithm. Branch lengths are not to scale in order to highlight the topology of the tree. The numbers below the nodes represent statistical support. Whole italicized numbers represent bootstrap support from the maximum-likelihood tree (not shown); decimal numbers that are not italicized represent Bayesian posterior probability

To trace the historical biogeography during the evolution of the Glyptosternoids, the ancestral distribution of the internal nodes were reconstructed with the Dispersal-Extinction-Cladogenesis (DEC) model [55] using the RASP program v. 3.02. [56] based on the ML tree inferred from the data from 12 mitochondrial protein-coding genes (Fig. 2) and the distribution pattern (Additional file 1: Table S3) of Sisoridae.

### Divergence time estimation

**Fig. 3:**
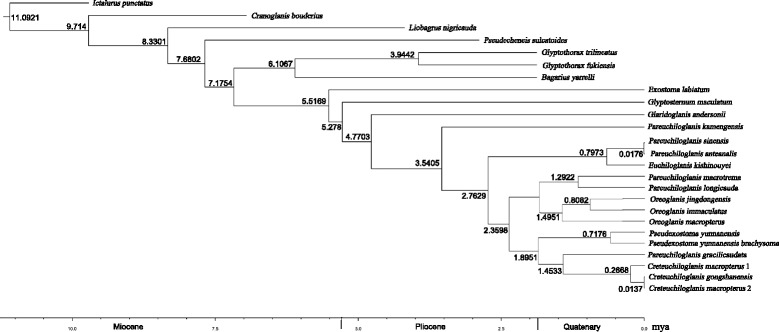
Time tree for the Chinese Sisoridae. The tree topology derived from these fish is generally consistent with the Bayesian inference shown in Fig. 2 (with the exception of *Exostoma labiatum* and *Glyptosternum maculatum*). Branch lengths are proportional to divergence times. The numbers at the right of the nodes are the estimates of the mean divergence times (in Mya)

The divergence times of the Chinese Glyptosternoids were determined using the protein-coding genes with the Bayesian molecular dating program Beast [57], according to the manual for the program. Estimates were calibrated using two age constraints (C1 and C2; Fig. 3). The C1 calibration point is based on the fossil record of *Bagarius yarrelli* from the Pliocene (5.3–1.8 Ma ago) of the Siwalik Hills in India [37]. C2 represents an upper bound of 4 Ma, derived from the capture of the Tsangpo by the Brahmaputra River, which occurred prior to about this time [32]. These time estimates were conducted using the GTR + G + I model. Following a burn-in of the initial 25 % cycles, divergence times were sampled once every 1000 generations from 10^8^ Markov Chain Monte Carlo (MCMC) iterations. Convergence of the chains to a stationary distribution was checked by visual inspection using TRACER v1.4 [58]. We repeated this analysis twice with different MCMC conditions and confirmed the stability of our estimates.

### Substitution rate estimation and selection analyses

To estimate lineage-specific evolutionary rates for each branch of the Glyptosternoids and their closest relatives, the CODEML program in the PAML package [59] with the free-ratio model (model = 1) was run on each of the 12 protein-coding mitochondrial genes. The parameters dN, dS, dN/dS, N*dN, and S*dS values were obtained for each branch of the tree (Fig. 2), and genes were discarded if N*dN or S*dS were less than 1 or dS was greater than 1, according to a previous study [60].

In molecular evolutionary biology, the natural selection acting on protein-coding genes is often characterized by comparison of synonymous and nonsynonymous substitution rates [38]. The nonsynonymous over synonymous substitution rates, ω = dN/dS, is a widely used indicator for measuring the direction and magnitude of selective pressure on amino acid replacements. The ω values <1, =1, and >1 represent purifying selection, neutral evolution, and positive selection, respectively [61, 62]. The ω ratios of the mitochondrial genomes were estimated with the CODEML program of PAML [59] using the concatenated sequence of the 12 protein-coding genes (excluding ND6). To detect positive selection in the limited codon sites in particular lineages across the phylogenetic tree, we applied the branch-site model [61]. To confirm that the ω ratios of the positively selected sites (ω_pss_) were significantly higher than 1, we performed the likelihood ratio test (LRT) with the null hypothesis that the ω_pss_ value was 1. The candidates of the positively selected sites were predicted by the Empirical Bayes method [62].

### Hydrological and geological events pertinent to this study

It has long been recognized that paleo-drainages of major continental East Asian Rivers, draining the southeastern Tibet plateau margin, differed markedly from their current drainage patterns [32, 63–69]. Clark et al. [32] suggested that these rivers were once tributaries to a single southward flowing system, which drained into the South China Sea. Subsequent reorganization into modern major river drainages was primarily caused by river capture and reversal events associated with the initiation of Miocene uplifts in eastern Tibet [32]. Although large-magnitude tectonic shear, prompted by the In-dian–Asian collision around the eastern Himalayan syntaxis, river capture and reversal events cannot be ruled out as an additional factor influencing these large-scale changes in drainage patterns [32, 66]. As reviewed by Ruber et al. [27], the evolution of drainage systems in Asia can be summarized in four stages [32]. (a) Upper Yangtze, Middle Yangtze, Upper Me-kong, and Upper Salween rivers drained into the South China Sea through the paleo Red River. (b) Capture/reversal of the Middle Yangtze river redirected drainage away from the Red River and into the East China Sea through the Lower Yangtze river. (c) Capture of the Upper Yangtze River into the Lower Yangtze River, and of the Upper Mekong and Upper Salween rivers into their modern drainage position. The Tsangpo River was also captured to the south through the Irrawaddy River. (d) Capture of the Tsangpo river through the Brahmaputra river into its modern drainage position.

## Results

### Phylogenetic reconstruction

Saturation tests [70] that included all taxa found no evidence for saturation in the twelve protein gene tests (except ND6) and the RNA gene tests. In each case, the index of substitution saturation (Iss) was significantly less than the critical value (Iss.c; see [70]).

The results of the Bayesian and ML nucleotide analyses produced by MrBayes and RAxML for the 12 protein-coding gene sequence datasets showed a marked consistency in topological congruence, differing only in the support values for certain nodes (Fig. 2). Just like the results based on the concatenation datasets from the 12 mitochondrial protein-coding and 2 rRNA genes (see Additional file 1: Figs. S1 and S2), the phylogenies indicate monophyly of the Chinese Sisoridae (including *Pseudecheneis*, *Bagarius*, *Glyptothorax* and Glyptosternoids) with very high support values (PP = 1.00 and BP = 95, Fig. 2). Likewise, monophyly of glyptosternoids (including *Glyptosternum*, *Glaridoglanis*, *Exostoma*, *Euchiloglanis*, *Pseudexostoma*, *Oreoglanis*, *Pareuchiloglanis*, and *Creteuchiloglanis*) are recovered with great posterior probability (PP = 1.00 and BP = 100, Fig. 2) The Glyptosternoids and non-Glyptosternoids (*Bagarius*, *Glyptothorax*) form a sister group with high support values (Fig. 2). *Exostoma labiatum* was placed with other Glyptosternoids to form a sister group to *Glyptosternum maculatum* in the phylogenetic trees. However, *G. maculatum* was placed with other Glyptosternoid fishes to form a sister group to *E. labiatum* in the phylogenetic trees (Fig. 3). Specialized Glyptosternoids diverged into three main lineages: The first lineage included *Euchiloglanis kishinouyei* and *Pareuchiloglanis* from the Upper Yangtze (*P. anteanalis* and *P. sinensis*); the second included the *Creteuchiloglanis* from Nujiang, *Pareuchiloglanis gracilicaudata* and *Pseudexostoma*; and the third lineage included the *Oreoglanis*, *Pareuchiloglanis longicauda* and *P. macrotrem*. The latter two lineages form sister groups separate from the Upper Yangtze lineage. *Pareuchiloglanis* was not resolved as monophyletic.

Consistent topologies were found among the MrBayes phylogenies using only protein-coding genes (Fig. 2) as well as using concatenating rRNA and the 12 protein-coding genes by gene region partitioning. We therefore used the 12 protein gene sequences in our phylogenetic reconstructions.

### Divergence times among the Glyptosternoid fishes

Our newly estimated divergence times based on the mitochondrial genomes are shown in Fig. 3. Chinese Sisoridae were found to originate in the Late Miocene (c. 7.7 Ma), the Glyptosternoids later in the Late Miocene (c. 5.5 Mya), and the specialized Glyptosternoids, *Pareuchiloglanis*, *Oreoglanis*, *Creteuchiloglanis* and *Pseudexostoma*, between the Pleistocene and Holocene. These results also show that explosive speciation of the specialized Glyptosternoids occurred between the late Pliocene and the Quaternary (c. 2.8 Ma).

### Ancestral reconstruction of the geographical distribution

**Fig. 4:**
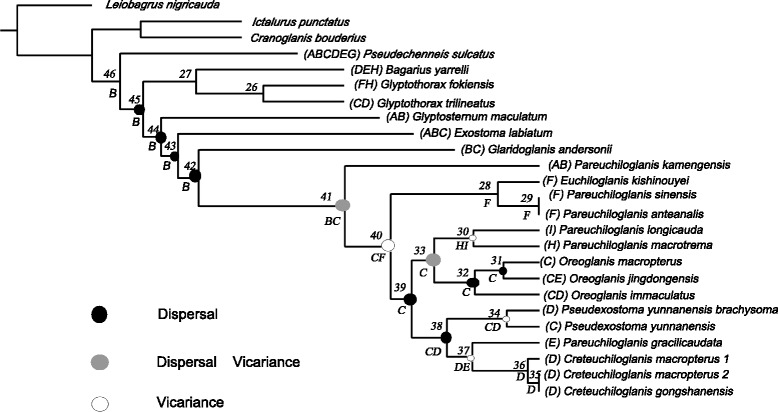
Results from the Dispersal-Extinction-Cladogenesis (DEC) analysis using the software RASP (Yu et al. [56]). Nodes of interest are marked by circles, assigned a unique number, and shaded according to the divergence process assigned. In this context, circles in gray represent nodes in which both vicariant and dispersal events were assigned. Note that incomplete lineage sorting and uncertainty regarding the ancestral node distribution reduce the robustness of the assignment. Letters below circles represent the distribution range with the highest probability; refer to the text for discussion on the robustness of these inferences

The optimal distributions at each ancestral node are given in Fig. 4. The analysis suggests basal lineages for the Glyptosternoid in the Tsangpo drainages (node 44). All the ancestors of basal Glyptosternoids lineages (nodes 42, 43, and 44) were distributed in the Tsangpo basin and then spread into drainages of the Tibetan Plateau. The reconstruction of the ancestral distribution ranges in certain deeper nodes is expected to be less robust due to the higher number of ranges among daughter lineages, especially for node 41 (Additional file 1: Table S4).

### Accelerated evolution in the lineage of Glyptosternoid fish

**Fig. 5:**
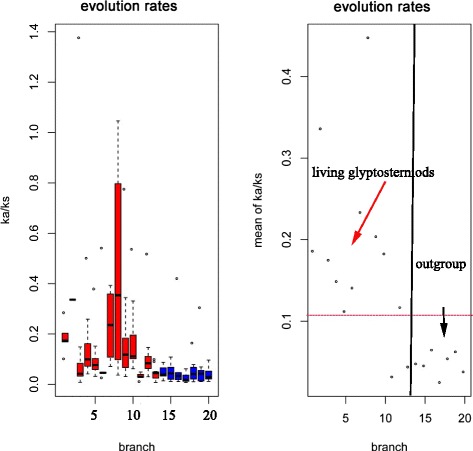
Evolutionary rates of Glyptosternoids. The species of each branch are listed in Additional file 1: Table S5: branches of the Glyptosterniods in Fig. 5

Averaged across all 12 protein-coding genes, the ratio of nonsynonymous to synonymous substitutions is significantly higher in most of the Glyptosternoids lineages than observed in other non-Glyptosternoid lineages (Additional file 1: Table S5), suggesting accelerated function evolution in the Glyptosternoids lineages (Fig. 5). In addition, the basal species of Glyptosternoids had lower ratios of nonsynonymous to synonymous substitutions than the specialized species (Fig. 5a).

### Positively selected genes in the mitochondrial genome

**Fig. 6:**
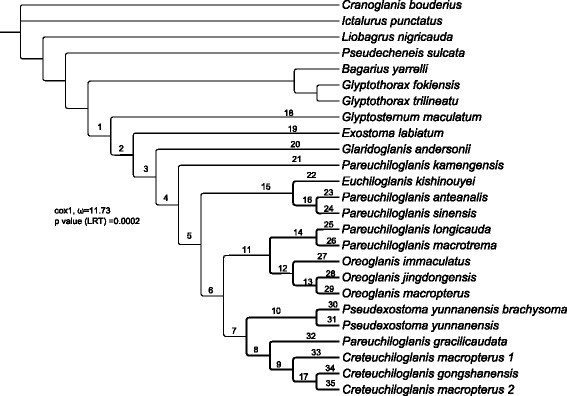
Detection of positively selected codon sites in the complete mitochondrial protein genes (excluding ND6). The MrBayes tree topology (Fig. 2) was used for this analysis. “ω_pss_” represents the ω value of positively selected sites. “*P*-value (LRT)” represents the *p*-value of the likelihood ratio test of the alternative hypothesis (ω_pss_ = MLE) and the null hypothesis (ω_pss_ = 1). The branches in which positively selected codon sites were detected are indicated by bold black lines

Using the branch-site model, positively selected signals were detected in fifteen branches. Among them, the signals of eight branches (branches 7, 8, 12, 13, 23, 24, 26, and 29) were weak, and AIC preferred the null hypothesis (ω_pss_ = 1). Accordingly, the likelihood of positive selection in these eight branches was negligible. In contrast, AIC preferred the alternative hypothesis (ω_pss_ = maximum likelihood estimation) for the other seven branches (branches 1, 10, 16, 21, 22, 25, and 31; Fig. 6 and Additional file 1: Table S3). Branch 1 represents the common ancestral branch of the Chinese Glyptosternoids. According to our estimates, ω_pss_ was 10.73; thus, the ω_pss_ value was significantly higher than 1 (the *p*-value was 0.0002). Positively selected sites occurred in the COX1 gene. The candidates of the positively selected sites are shown in Additional file 1: Table S6. In the other six branches, which represent specialized Glyptosternoids, positively selected sites occurred in most of the mitochondrial protein-coding genes (Additional file 1: Table S6).

## Discussion

### Origin and expansion of Chinese Glyptosternoid fishes and the southeastern Tibetan drainage patterns

The evolutionary time scale of Glyptosternoids diversification is still a controversial issue. Our estimates are much younger than those of Peng [28] and Guo [23], both of which were based on several gene sequences (such as cytb) and limited sample sizes, especially with respect to the strict clock method used by Guo [23]. This work incorporated far more Glyptosternoid samples, especially *Pareuchiloglanis*, *Oreoglanis* and *Creteuchiloglanis*. Thus, the Glyptosternoids most likely originated in the Late Miocene and radiated during the Pliocene and Quaternary.

Based on our biogeographic results, we believe that the third explanation (see section *introduction*) agrees best with our phylogeny (Fig. 2). An ancestral Glyptosternoids species was widely distributed throughout the Brahmaputra drainage in the eastern Himalayas and Tibetan area during the Late Miocene (c. 7.7 Ma). The eastern Himalayas areas included what are now Nepal, India, Bhutan and China (Tibet and Yunnan). Then, the Glyptosternoids dispersed into the eastern Tibetan drainages and species evolved specialized adaptive traits suited to rapidly flowing water habitats, such as depressed bodies and heads, smaller gill openings and pinniform rugae on the rims of paired fins (such as pectoral fins). The teeth of these species also diversified, and their feeding habits changed (pointed-form teeth: fish and arthropods; shovel-form teeth: algae; *Exostoma*-like teeth: algae and arthropods). A rapid uplift of the Tibetan Plateau occurred at approximately 3.6 Ma, and the Yangtze, Nujiang, Lancang and Yuanjiang Rivers formed at this time. The specialized species, *Pareuchiloglanis, Creteuchiloglanis, Euchiloglanis, Oreoglanis* and *Pseudexostoma*, originated during this phase. These species then spread to the downstream portions of the Nujiang, Lancangjiang, Yaluzangbujiang (Brahmaputra) and Irrawaddy Rivers to form the current day distribution pattern, yielding a great species diversity. The process of speciation among Chinese Glyptosternoids resulted from dispersal and vicariance events associated with the uplift of the Tibetan area and the newly formed river systems.

The historical biogeography of a lineage reflects aspects of the history of the region in which the species, or lineage, is distributed. We reconstructed a possible scenario for biogeographic history of the southeastern Tibet inferred from the geographical distribution of the Chinese Glyptosternoids across this drainage area. An exact search with DEC suggested basal lineages for the Glyptosternoids in the Tsangpo drainages (Fig. 4). The inferred ancestral areas (based on DEC results) are shown in Fig. 4. To distinguish between these scenarios, geological evidence and the likelihood of widespread ancestors must be considered. The use of a wide range of calibrated rates allows us to compare the molecular divergence times with the available data on geological events in this area and to test various vicariance/dispersal hypotheses. Vicariance theorists assume that common distributional patterns result from shared vicariance events. The hypothesis of a vicariant event between the formerly connected Tsangpo and Upper Irrawaddy is also supported by data from Badidae species [27].

Our results seem to agree with the geological evidence for the separation of these drainages due to tectonic uplift in eastern Tibet. The geological events appear to have played a primary role in the diversification of Chinese Glyptosternoids. In southeastern Tibet, the order of the appearance of the specialized species represents the order in which the rivers became isolated [26]. The rivers were once tributaries of a single southward-flowing system that drained into the South China Sea [31, 32]. In the early Pliocene, the Tsangpo and Brahmaputra Rivers became isolated from the ancient Red River. Until the middle Pliocene, the Jinsha River was isolated and fostered specialized diversification at the genus level; these fish then expanded into the rivers of Yunnan and Sichuan during the most recent uplift episode in the Himalayan area. The modern Red River and Pearl River became isolated in the late Pliocene. At this time, the Glyptosternoids spread into the Nujiang and Lancang Rivers. In the Pleistocene, the Lancangjiang River was isolated following the vicariance between the modern Red River and Pearl River. In the Holocene, the Nujiang River separated from the Irrawaddy River. These results agree with [32]. River capture and reversal occurred during the rapid uplift of the Tibetan Plateau [32]. The origin and expansion of Chinese Glyptosternoid fishes were affected not only by the three cycles of uplift and two large-scale peneplanation events of the Qinhai-Tibetan Plateau but also the river capture and reversal events in eastern Tibet in the Miocene due to the uplift of the Qinghai-Tibetan Plateau. Both factors have influenced the modern drainage patterns in eastern Tibet.

### Mitochondrial adaptive genetic basis for high-elevation living

Adaptive evolution may preferentially occur at the molecular level and may be expressed as an increased ratio of nonsynonymous substitutions to synonymous substitutions [71]. Our study adds to the growing body of evidence for adaptive evolution in the mitochondrial genome of high-elevation species. Similar to previous studies of major adaptations to high-elevation habitats of different endothermic animals based on genomic data [8, 9, 11], the Glyptosternoid fish lineage exhibits accelerated evolution in the mitochondrial genome relative to other non-Glyptosternoid fish lineages. A consequence of the fact that species living in similar ecological environments can be shaped by convergent evolution to form physiological or morphological similarities [72]. In particular, the specialized Glyptosternoid fishes have higher nonsynonymous to synonymous substitutions than the basal species, suggesting the specialized species developed accelerated evolutionary rates in order to adapt to the high-elevation environment. Thus, the mitochondrial genes of Glyptosternoid fishes may have experienced adaptively accelerated evolutionary rates to better adapt to the extreme environments of the Tibetan Plateau because accelerated evolution is usually driven by positive selection.

We identified signatures of positive selection in the branch of the Chinese Glyptosternoid fish, and these signatures may indicate adaptation to physiological hypoxia and cold stress. Branch 1 (Fig. 6) represents the common ancestral branch of the Glyptosternoid fishes in China. According to our estimates, ω_pss_ was 10.73, i.e., significantly higher than 1 (*p*-value 0.0002), for the gene COX1 under positive selection. These findings are similar to previous studies on native high-elevation animals that found that the COX1 gene experienced positive selection in Tibetan antelope [16] and plateau pikas [19]. In cold environments, a less efficient OXPHOS is preferred because it results in maximum heat generation and minimum ATP and ROS production [73]. Mitochondria produce increased quantities of NO under hypoxia. Cytochrome *c* oxidase has been identified as the mitochondrial enzyme that reduces NO^-2^ to NO, which induces expression of nuclear hypoxic genes, possibly via a pathway that involves protein nitration [74]. Therefore, the modification of the structure and/or activity of cytochrome *c* oxidase or complex IV of the respiratory chain may contribute to hypoxia adaptation. Furthermore, because oxygen is the ultimate electron acceptor, which results in the production of H_2_O in a process catalyzed by cytochrome *c* oxidase, modifications of the cytochrome *c* oxidase activities are expected to facilitate coping with a reduced oxygen supply. These modifications would affect mitochondrial NO production and, consequently, hypoxic signaling. Considering the reconstructed ancestral geographical distribution areas, this positive selection likely occurred during the process of high-elevation adaptation.

The other branches’ candidates for positively selected sites were predicted by the Empirical Bayes method [62] and are shown in Additional file 1: Table S6. Branch 21, branches 16 and 22, branch 25, branch 10 and branch 31 appear to have been distributed in the Brahmaputra/Tsangpo, Upper Yangtze, Pearl, Irrawaddy and Salween Rivers, respectively. Branch 21, which includes *Pareuchiloglanis kamengensis*, is found in the Brahmaputra/Tsangpo drainages and experienced positive selection. In this branch, the proportion of positively selected sites was 0.97 %, which corresponds to 35 codon sites in different genes. These codon sites are composed of Complex I, Complex III, Complex IV and Complex V. Branches 10, 16, 22, and 25, which were distributed in Irrawaddy, Salween, Upper Yangtze, and Pearl Rivers, were also under positive selection and correspond with many codon sites. However, these codon sites do not contain the genes associated with Complex V. Polypeptides are all subunits of the oxidative phosphorylation (OXPHOS) enzyme complexes. In aerobic organisms, OXPHOS supplies most of the ATP needed for cell metabolism. During this process, electrons from NADH or FADH2 are transferred to O_2_ via a series of electron carriers, which pump protons through the inner mitochondrial membrane, generating a proton gradient that drives ATP synthesis via ATP synthase (Complex V). Complex V consists of two main structural domains: an intrinsic membrane domain (F0) and an extrinsic globular domain (F1), linked by a central and a peripheral stalk [75]. The mammalian mitochondrial ATP synthase comprises at least 16 subunit types [76], among which the mitochondrial-encoded ATP6 and ATP8 are essential subunits [77]. In branch 21 (*Pareuchiloglanis kamengensis*), there are one and three positively selected candidate sites corresponding to ATP 6 and ATP 8, respectively (Additional file 1: Table S6).

Taking into account the reconstructed ancestral states of the geographical distribution (Fig. 4) and positively selected codon sites (Fig. 6), we conclude that the common ancestral branch of the Glyptosternoid fishes was distributed across the Qinghai-Tibet Plateau in China and adapted to physiological hypoxia and cold stress through transforming the codon sites in COX1. These findings are similar to those suggested for the Tibetan antelope [16] and plateau pika [19] but differ from findings reported for artiodactyls, perissodactyls, snub-nosed monkeys and humans living in high-elevation environments [2, 3, 18, 20]. Different adaptive strategies have likely been developed by different lineages. Following the dispersion of the Glyptosternoid fish into the drainages surrounding southeastern Tibet, the changes to additional codon sites corresponded to other mitochondrial genes associated with Complex I, Complex III, Complex IV and Complex V.

## Conclusions

We reconstructed a possible scenario for the southeastern Tibetan drainage patterns based on the adaptive geographical distribution of the Chinese Glyptosternoids in this drainage. In addition, the Glyptosternoid fishes lineage had a higher ratio of nonsynonymous to synonymous substitutions than those found in non-Glyptosternoids. They may have experienced accelerated evolutionary rates in mitochondrial genes that were driven by positive selection to better adapt to the high-elevation environment of the Tibetan Plateau.

### Availability of supporting data

The data set supporting the results of this article is available in the GenBank under KP872682, KP872683, KP872690-KP872697 and provided as supplementary data.

## Additional file

Additional file 1: Figure S1. Phylogenetic tree estimated using the MrBayes algorithm with 12 protein-coding genes and 2 rRNA genes. Branch lengths are not to scale to highlight the topology of the tree. Numbers below nodes represent Bayesian posterior probability. **Figure S2.** Phylogenetic tree estimated using the maximum-likelihood method with 12 protein-coding genes and 2 rRNA genes. Numbers below nodes represent bootstrap support from the maximum-likelihood tree. **Table S1.** Location of samples and accession numbers for all sequences used in this study. **Table S2.** characteristics of the original mitochondrial genome sequences of 10 species of Glyptosterniod fishes. **Table S3.** Know distribution of Chinese Sisoridae fishes in the study. A: Brahmaputra B: Yaluzangbujiang (Tsangpo), C: Irrawady, D: Nujiang (Salween), E: Lancangjiang (Mekong), F: Jinshajiang (Upper Yangtze), G: Ganges, H: Red River, I: Pearl River. **Table S4.** Probabilities in nodes for the ancestral states of the Glyptosterniods distribution areas with Dispersal-Extinction-Cladogenesis (DEC) analysis (Fig. 4). **Table S5.** branches of the Glyptosterniods in figure 5. **Table S6.** Candidate of the positively selected genes. (DOCX 533 kb)
