# Supplementary material for: Biogeographic history and high-elevation adaptations inferred from the mitochondrial genome of Glyptosternoid fishes (Sisoridae, Siluriformes) from the southeastern Tibetan Plateau
Source: BMC Evol Biol. 2015 Oct 28;15:233. doi: 10.1186/s12862-015-0516-9 (PMC4625616; doi:10.1186/s12862-015-0516-9)
Supplement: Additional file 1: Figure S1. — Phylogenetic tree estimated using the MrBayes algorithm with 12 protein-coding genes and 2 rRNA genes. Branch lengths are not to scale to highlight the topology of the tree. Numbers below nodes represent Bayesian posterior probability. Figure S2. Phylogenetic tree estimated using the maximum-likelihood method with 12 protein-coding genes and 2 rRNA genes. Numbers below nodes represent bootstrap support from the maximum-likelihood tree. Table S1. Location of samples and accession numbers for all sequences used in this study. Table S2. characteristics of the original mitochondrial genome sequences of 10 species of Glyptosterniod fishes. Table S3. Know distribution of Chinese Sisoridae fishes in the study. A: Brahmaputra B: Yaluzangbujiang (Tsangpo), C: Irrawady, D: Nujiang (Salween), E: Lancangjiang (Mekong), F: Jinshajiang (Upper Yangtze), G: Ganges, H: Red River, I: Pearl River. Table S4. Probabilities in nodes for the ancestral states of the Glyptosterniods distribution areas with Dispersal-Extinction-Cladogenesis (DEC) analysis (Fig. 4). Table S5. branches of the Glyptosterniods in figure 5. Table S6. Candidate of the positively selected genes. (DOCX 533 kb) [file 12862_2015_516_MOESM1_ESM.docx]

**Title**: Biogeographic history and high-elevation adaptations inferred from the mitochondrial genome of Glyptosternoid fishes (Sisoridae, Siluriformes) from the southeastern Tibetan Plateau

**Authors**: XIUHUI MA^1,2^, JINGLIANG KANG^2,3^, WEITAO CHEN^2,3^, CHUANJIANG, ZHOU^2^, and SHUNPING HE^2^

1 School of Life Science, Southwest University, Beibei, Chongqing, 400715, China

2 The Key Laboratory of Aquatic Biodiversity and Conservation of Chinese Academy of Sciences, Institute of Hydrobiology, Chinese Academy of Sciences, Wuhan, Hubei, 430072, China

3 University of Chinese Academy of Sciences, Beijing, 10001, China

Email: XIUHUI MA: lovemxh@126.com;

JINGLIANG KANG: [jlkang@ihb.ac.cn](mailto:jlkang@ihb.ac.cn);

WEITAO CHEN: [chenweitao@ihb.ac.cn](mailto:chenweitao@ihb.ac.cn);

CHUANJIANG, ZHOU: [chuanjiang88@163.com](mailto:chuanjiang88@163.com).

Correspondence: Shun-Ping He; Address: The Key Laboratory of Aquatic Biodiversity and Conservation of Chinese Academy of Sciences, Institute of Hydrobiology, Chinese Academy of Sciences, Wuhan, Hubei, 430072, China; Fax: +86-27-68780071; E-mail: [clad@ihb.ac.cn](mailto:clad@ihb.ac.cn)

**
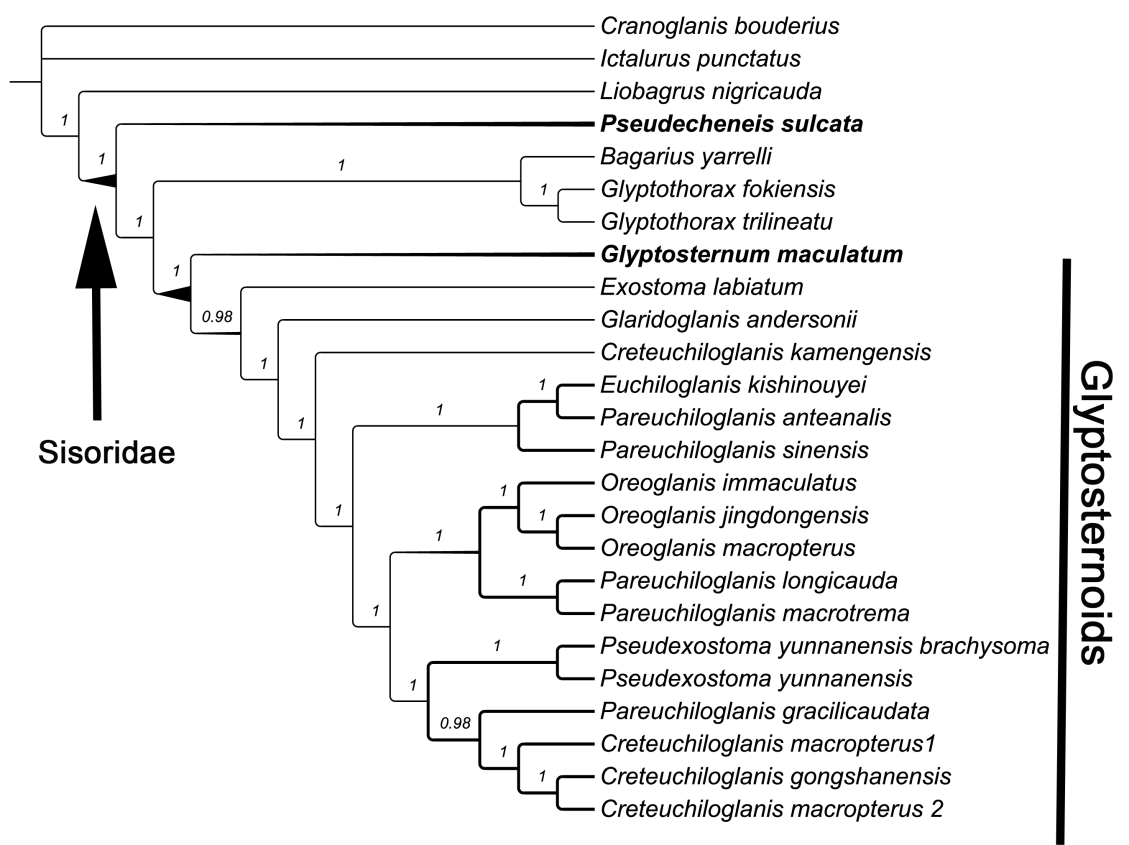
**

**Figure S1**. Phylogenetic tree estimated using the MrBayes algorithm with 12 protein-coding genes and 2 rRNA genes. Branch lengths are not to scale to highlight the topology of the tree. Numbers below nodes represent Bayesian posterior probability.


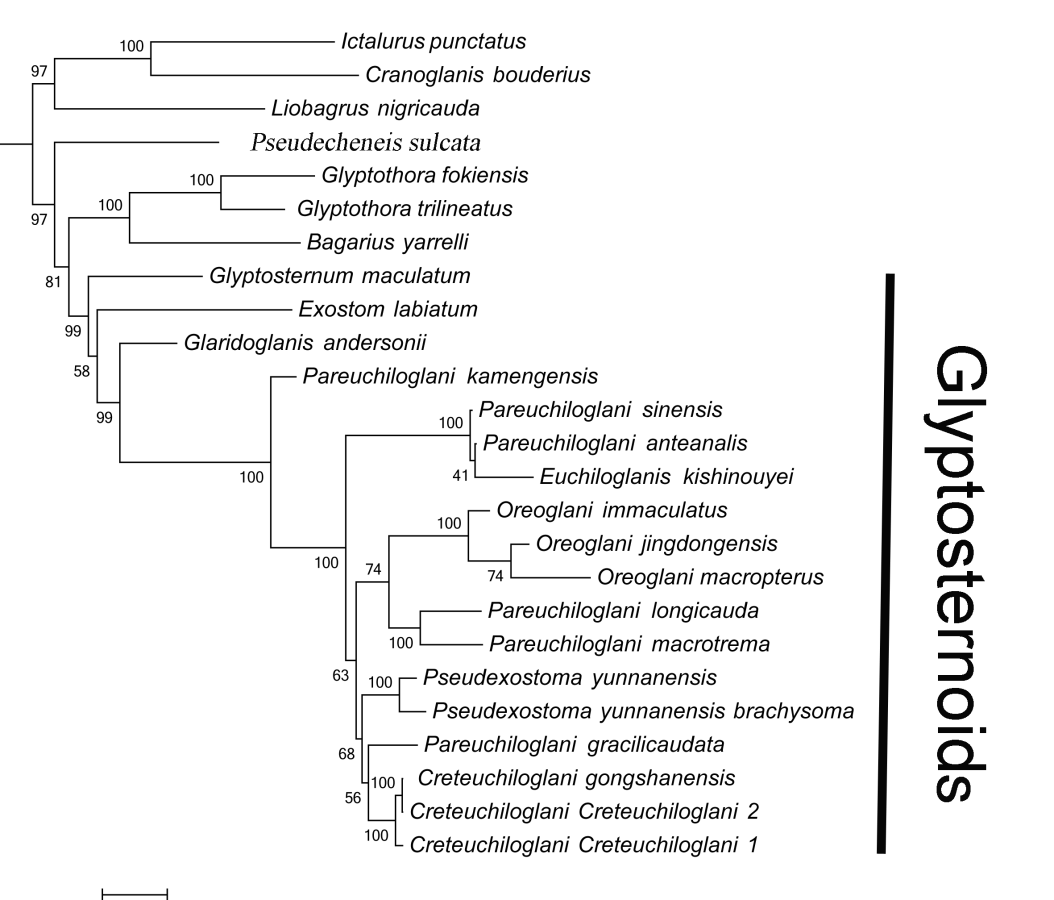


**Figure S2**. Phylogenetic tree estimated using the maximum-likelihood method with 12 protein-coding genes and 2 rRNA genes. Numbers below nodes represent bootstrap support from the maximum-likelihood tree.

**Table S1:** Location of samples and accession numbers for all sequences used in this study

| Taxon | location | Accession number |
| --- | --- | --- |
| *Creteuchiloglanis macropterus 1* | Baoshan, Yunnan | KP872682 |
| *Creteuchiloglanis macropterus 2* | Fugong, Yunnan | KP872683 |
| *Creteuchiloglanis gongshanensis* | Gongshan, Yunnan | KP872697 |
| *Pseudexostoma yunnanensis brachysoma* | Baoshan, Yunnan | KP872696 |
| *Oreoglanis immaculatus* | Tengchong, Yunnan | KP872690 |
| *Oreoglanis jingdongensis* | Tengchong, Yunnan | KP872691 |
| *Pareuchiloglanis macrotrema* | Yuan River, Yunnan | KP872694 |
| *Pareuchiloglanis longicauda* | Nanpan River, Yunnan | KP872693 |
| *Pareuchiloglanis sinensis* | Daduhe, Sichuan | KP872695 |
| *Pareuchiloglanis anteanalis* | Daduhe, Sichuan | KP872692 |
| *Glyptosternum maculatum* | - | NC_021597.1 |
| *Glaridoglanis andersonii* | - | NC_021600.1 |
| *Exostoma labiatum* | - | NC_021601.1 |
| *Euchiloglanis kishinouyei* | - | NC_021598.1 |
| *Pareuchiloglanis gracilicaudata* | - | NC_021603.1 |
| *Pareuchiloglanis kamengensis* | - | NC_021599.1 |
| *Pseudexostoma yunnanensis* | - | NC_021604.1 |
| *Oreoglanis macropterus* | - | NC_021607.1 |
| *Pseudecheneis sulcatoides* | - | NC_021605.1 |
| *Bagarius yarrelli* | - | NC_021606.1 |
| *Glyptothorax fukiensis* | - | NC_018769.1 |
| *Glyptothorax trilineatus* | - | NC_021608.1 |
| *Liobagrus nigricauda* | - | NC_021407.1 |
| *Cranoglanis bouderius* | - | NC_008280.1 |
| *Ictalurus punctatus* | - | NC_003489.1 |

**Table S2:** characteristics of the original mitochondrial genome sequences of 10 species of Glyptosterniod fishes

| Species | Raw data | Clean data | Contig | N50 | Max contig |
| --- | --- | --- | --- | --- | --- |
|  | (pair-end reads number) | (pair-end reads number) | (number) | (bp) | (bp) |
| *Creteuchiloglanis macropterus* 1 | 618, 005 | 519, 595 | 545 | 390 | 16, 607 |
| *Creteuchiloglanis macropterus* 2 | 592, 603 | 499, 532 | 2014 | 358 | 33, 178 |
| *Creteuchiloglanis gongshanensis* | 608, 440 | 525, 305 | 415 | 578 | 33, 173 |
| *Pseudexostoma yunnanensis brachysoma* | 683, 761 | 596, 898 | 1457 | 470 | 10, 536 |
| *Oreoglanis immaculatus* | 629, 295 | 541, 047 | 2401 | 302 | 9, 510 |
| *Oreoglanis jingdongensis* | 623, 323 | 543, 762 | 953 | 430 | 33, 136 |
| *Pareuchiloglanis macrotrema* | 1, 116, 053 | 1, 010, 786 | 2723 | 427 | 33, 164 |
| *Pareuchiloglanis longicauda* | 571, 252 | 390, 233 | 4010 | 297 | 10, 783 |
| *Pareuchiloglanis sinensis* | 739, 393 | 622, 964 | 2092 | 322 | 13, 380 |
| *Pareuchiloglanis anteanalis* | 2, 096, 911 | 1, 851, 774 | 3567 | 319 | 13, 670 |

**Table S3:** Know distribution of Chinese Sisoridae fishes in the study. A: Brahmaputra B: Yaluzangbujiang (Tsangpo), C: Irrawady, D: Nujiang (Salween), E: Lancangjiang (Mekong), F: Jinshajiang (Upper Yangtze), G: Ganges, H: Red River, I: Pearl River.

| No. | Taxon | A | B | C | D | E | F | G | H | I |
| --- | --- | --- | --- | --- | --- | --- | --- | --- | --- | --- |
| 1 | *Pseudecheneis sulcatoides* | ● | ● | ● | ● | ● |  | ● |  |  |
| 2 | *Bagarius yarrelli* |  |  |  | ● | ● |  |  | ● |  |
| 3 | *Glyptothorax fukiensis* |  |  |  |  |  | ● |  | ● |  |
| 4 | *Glyptothorax trilineatus* |  |  | ● | ● |  |  |  |  |  |
| 5 | *Glyptosternum maculatum* | ● | ● |  |  |  |  |  |  |  |
| 6 | *Exostoma labiatum* | ● | ● | ● |  |  |  |  |  |  |
| 7 | *Glaridoglanis andersonii* |  | ● | ● |  |  |  |  |  |  |
| 8 | *Euchiloglanis kishinouyei* |  |  |  |  |  | ● |  |  |  |
| 9 | *Pareuchiloglanis kamengensis* | ● | ● |  |  |  |  |  |  |  |
| 10 | *Creteuchiloglanis gongshanensis* |  |  |  | ● |  |  |  |  |  |
| 11 | *Creteuchiloglanis macropterus 1* |  |  |  | ● |  |  |  |  |  |
| 12 | *Creteuchiloglanis macropterus 2* |  |  |  | ● |  |  |  |  |  |
| 13 | *Pareuchiloglanis longicauda* |  |  |  |  |  |  |  |  | ● |
| 14 | *Pareuchiloglanis macrotrema* |  |  |  |  |  |  |  | ● |  |
| 15 | *Pareuchiloglanis sinensis* |  |  |  |  |  | ● |  |  |  |
| 16 | *Pareuchiloglanis anteanalis* |  |  |  |  |  | ● |  |  |  |
| 17 | *Pareuchiloglanis gracilicaudata* |  |  |  |  | ● |  |  |  |  |
| 18 | *Oreoglanis immaculatus* |  |  | ● | ● |  |  |  |  |  |
| 19 | *Oreoglanis macropterus* |  |  | ● |  |  |  |  |  |  |
| 20 | *Oreoglanis jingdongensis* |  |  | ● |  | ● |  |  |  |  |
| 21 | *Pseudexostoma yunnanensis* |  |  | ● | ● |  |  |  |  |  |
| 22 | *Pseudexostoma yunnanensis brachysoma* |  |  |  | ● |  |  |  |  |  |

**Table s4:** Probabilities in nodes for the ancestral states of the Glyptosterniods distribution areas with Dispersal-Extinction-Cladogenesis (DEC) analysis (fig 4)

| node28 | node29 | node30 | node31 | node32 | node33 | node34 | node35 | Node36 |
| --- | --- | --- | --- | --- | --- | --- | --- | --- |
| F:1.0000 | F:1.0000 | HI:0.8183 | C:1.000 | C:0.7568 | C:0.5332 | CD:1.0000 | D:1.0000 | D:1.0000 |
|  |  | *:0.1817 |  | *:0.2432 | *:0.4668 |  |  |  |
| Node37 | Node38 | Node39 | Node40 | Node41 | Node42 | Node43 | Node44 | Node45 |
| DE:0.7491 | CD:0.5872 | C:0.7306 | CF:0.4057 | BC:0.2021 | B:0.6991 | B:0.7140 | B:0.7354 | B:0.6785 |
| *:0.2509 | *:0.4128 | *:0.2694 | *:0.5943 | * :0.7979 | *:0.3009 | *:0.2860 | *:0.2646 | *:0.3215 |

**Table s5:** branches of the Glyptosterniods in figure

| branch | Taxon | ka/ks(mean) |
| --- | --- | --- |
| 1 | *Creteuchiloglanis macropterus 1* | 0.1858 |
| 2 | *Creteuchiloglanis gongshanensis* | 0.3358 |
| 3 | *Pareuchiloglanis gracilicaudata* | 0.1748 |
| 4 | *Pseudexostoma yunnanensis brachysoma* | 0.1486 |
| 5 | *Pseudexostoma yunnanensis* | 0.1118 |
| 6 | *Pareuchiloglanis macrotrema* | 0.1407 |
| 7 | *Pareuchiloglanis longicauda* | 0.2332 |
| 8 | *Oreoglanis immaculatus* | 0.4474 |
| 9 | *Euchiloglanis kishinouyei* | 0.2037 |
| 10 | *Pareuchiloglanis kamengensis* | 0.1825 |
| 11 | *Glaridoglanis andersonii* | 0.0316 |
| 12 | *Exostoma labiatum* | 0.1169 |
| 13 | *Glyptosternum maculatum* | 0.0438 |
| 14 | *Bagarius yarrelli* | 0.0476 |
| 15 | *Glyptothorax trilineatus* | 0.0452 |
| 16 | *Glyptothorax fukiensis* | 0.0647 |
| 17 | *Pseudecheneis sulcatoides* | 0.0249 |
| 18 | *Liobagrus nigricauda* | 0.054 |
| 19 | *Cranoglanis bouderius* | 0.0624 |
| 20 | *Ictalurus punctatus* | 0.0379 |

**Table s6:** Candidate of the positively selected genes

| Branch | 1 | 10 | 16 | 21 | 22 | 25 | 31 |
| --- | --- | --- | --- | --- | --- | --- | --- |
| ω | 10.73 | 10.01201 | 84.25193 | 12.56049 | 999 | 998.9976 | 999 |
| p-value(LRT) | 0.0002 | 0.0438 | 0.0004 | 0.0009 | 0 | 0 | 0 |
| Gene:number | cox1 | cytb：2 | ND5：1 | ATP6:1 | ND5:1 | ND5:5 | ND1：3 |
|  |  | ND1：3 | ND4：1 | ND5:4 | ND4L:5 | ND4:4 | COX:1 |
|  |  | cox1:1 | ND3：4 | COX2:2 | ND3:19 | ND2:41 |  |
|  |  |  | COX3：3 | COX3:5 | COX3:12 | CYTB:3 |  |
|  |  |  | CYTB:2 | ND2:1 | cytb:1 | ND1:36 |  |
|  |  |  |  | CYTB:4 | ND1:1 |  |  |
|  |  |  |  | ND1:7 | COX1:2 |  |  |
|  |  |  |  | COX1:8 |  |  |  |
|  |  |  |  | ATP8:3 |  |  |  |
